# Maximizing waste heat recovery from a building-integrated edge data center

**DOI:** 10.1038/s41598-025-22498-x

**Published:** 2025-11-05

**Authors:** Mustafa Kuzay, Ender Demirel, Cagatay Yilmaz, Binod Prasad Koirala, Philipp Heer

**Affiliations:** 1 Design and Simulation Tech. Inc, 26480 Eskisehir, Türkiye; 2 Department of Civil Engineering, Eskisehir Osmangazi University, 26480 Eskisehir, Türkiye; 3 RISE Research Institutes of Sweden, Luleå, Sweden; 4 Urban Energy Systems Laboratory, Swiss Federal Laboratories for Materials Science and Technology (Empa), Überlandstrasse 129, Dübendorf, 8600 CH Switzerland

**Keywords:** Edge data center, Workload allocation, Heat recovery, CHT model, Energy science and technology, Engineering, Mathematics and computing

## Abstract

Small data centers can be integrated into the energy systems of commercial and tertiary buildings to capture waste heat generated by servers, mitigate environmental impacts and enhance energy efficiency. This study introduces a novel methodology for maximizing waste heat capture from the cooling coils by optimizing workload distribution in an edge data center consisting of air-cooled servers. The maximization of outlet temperatures algorithm (MOTA) was developed using a validated fast thermal evaluation approach. Experimental studies were conducted to characterize parameters of the cooling system such as air and water flow rates and effectiveness of the coil. A multi-region conjugate heat transfer (CHT) numerical model was developed to demonstrate feasibility of the proposed approach. Heat transfer through the cooling coil was numerically modeled using the effectiveness-NTU method. Good agreement was achieved between the simulated and measured water temperatures at the outlet of the cooling coil. Numerical simulations conducted using the validated CHT model show that the MOTA can improve heat recovery by up to 17.1% under various IT loads. Furthermore, optimizing the water flow rate can reduce the cooling load by up to 53.2%. These combined results highlight the potential of the proposed algorithm for energy-efficient management of data centers. Computational cost and real-world applicability of the proposed algorithm were discussed in detail.

## Introduction

The rapid advancements in information technologies (IT) have highlighted the crucial role played by data centers (DCs) in meeting increasing power demand in recent years. These facilities are associated with high energy usage and significant carbon emissions^1–4^. Effective cooling is essential for the reliable performance and longevity of IT equipment as it prevents overheating. Additionally, the waste heat generated by IT equipment offers a valuable opportunity for recovery. The excess heat can be utilized for heating buildings, reducing overall energy consumption and enhancing the sustainability of data center operations by integrating waste heat recovery systems. This dual approach improves energy efficiency while contributing to environmental sustainability and resource optimization^5–10^.

DCs serve as critical infrastructures within IT networks worldwide to power and cool IT equipment. The primary goal is to establish a network environment that operates efficiently and reliably^11^. These facilities range in complexity from a single rack in a server room to large-scale computing facilities consisting of diverse IT and cooling equipment designed to ensure continuous operation^12–14^. As a prominent example, a recent report by the Central Statistics Office (CSO) highlighted that Irish data centers consume 18% of the total energy supply, representing a 31% increase over the previous year. The operations of DCs also account for about a quarter of greenhouse gas emissions^14^ and are considered to have the fastest growing carbon footprint within the entire information and communications technologies (ICT) sector^15^. Such tremendous energy demand and carbon footprint by DCs have highlighted the importance of energy-aware management in recent years.

Globally, cooling accounts for about 40% of total energy consumption in DCs^16^. While air-cooling is still a widely used cooling method in DCs due to its practical options, liquid-cooling has emerged as an effective cooling for high power IT equipment due to the high heat transfer capacity. Modular air-cooled systems such as raised-floor setups, in-row cooling^17,18^, rear-door cooling^19^, and rack-mounted cooling^20^, can handle a maximum heat load of up to 20 kW per rack^21^. Various cooling methods have emerged as efficient and eco-friendly technologies to recover waste heat from DCs^22^. One potential alternative is on-chip liquid cooling, where the primary coolant is delivered directly to the main processor, GPU and ancillary circuits remove the heat generated by power equipment. Researchers have identified major competing chip cooling technologies such as microchannel single-phase flow, porous medium flow, jet impingement cooling and microchannel two-phase flow and pool boiling^23–26^. Despite recent advancements in cooling technologies, the majority of DCs today are air-cooled, meaning that the waste heat is released into the atmosphere, often using cooling tower or a chiller. Outlet temperatures of air-cooled servers are typically observed in the range of 35 to 45 °C, making it suitable for recovery and use to heat neighboring buildings. Consequently, waste heat recovery has emerged as a significant opportunity in DCs to reduce their environmental impact. In recent years, attention to waste-heat recovery from data centers has increased, reflecting both the rising energy demand of digital infrastructure and the need for sustainable integration with surrounding energy systems. Several technical and techno-economic studies have explored algorithms to improve recovery potential, focusing on methods to raise the quality and usability of waste heat and to enable its effective use in building services and district heating networks^27–33^.

Advances in computing technologies have established Computational Fluid Dynamics (CFD) models critical and promising tools for the thermal simulation of DCs. CFD models enable detailed analysis of flow and thermal structures in a data center to capture adverse effects such as recirculating hot zones and cold air by-pass^34–36^, which reduce efficiency and increase energy consumption by cooling. Among the CFD models, as a multi-region approach, Conjugate Heat Transfer (CHT) models offer valuable insights into the calculation of heat transfer based on the energy balance between distinct regions such as air, water and solid considering thermophysical features of each region. However, CHT models have been less used in the thermal modeling of DCs due to their complexities involved in generating multi-region mesh and definitions of boundary conditions that account for neighboring thermal regions. As CFD models require significant computational time to achieve a steady-state thermal map, fast prediction of internal thermal field is essential for the optimization of workloads under specific IT and cooling scenarios. Accordingly, the fast thermal evaluation (FTE) models are integrated to the energy-aware workload assignment algorithms to minimize IT energy consumption in DCs^37–39^. Typically, a series of CFD simulations conducted using referencing and profiling power consumptions to build airflow matrices that capture interactions between servers and recirculation effects. The literature has proposed thermal-aware and energy-efficient workload allocation strategies, such as temperature-aware scheduling and energy-minimization approaches. These works typically pursue two main objectives: (i) reducing hotspots to improve server reliability and cooling efficiency and (ii) lowering IT energy consumption by considering thermal maps and balancing workloads across servers. Therefore, the primary focus of the present study is to maximize waste heat recovery by optimizing workload traffic within the data center.

In this study, a three-dimensional CHT model was developed using open-source CFD libraries to simulate waste heat recovery from the cooling coils of an air-cooled micro data center. An experimental campaign was conducted under various operating scenarios and cooling conditions to characterize water flow rate through the cooling coils, the air flow rate supplied by the fans and the heat transfer across the cooling coils. The numerical model was validated with the experimental data collected through an integrated data monitoring and management platform. A novel workload allocation algorithm was designed by integrating the validated numerical model with the FTE to maximize waste heat recovery from building-integrated micro DCs. The results of numerical simulations, performed under various workload distributions and cooling conditions, were evaluated in terms of waste heat recovery and cooling power consumption. The rest of the paper is organized as follows: Sect. 2 introduces the pilot edge data center where the proposed algorithm is developed, tested and demonstrated. Section 3 presents experimental studies conducted for the characterization of the cooling system in the pilot data center. Section 4 presents the multi-region thermal model for the simulation of heat transfer between air and water. Section 5 focuses on the validation of the numerical model with the experimental data, demonstration of the performance of the proposed algorithm in terms of waste heat recovery and cooling power consumption. The last section discusses concluding remarks and future studies.

### Configuration of the data center

**Fig. 1:**
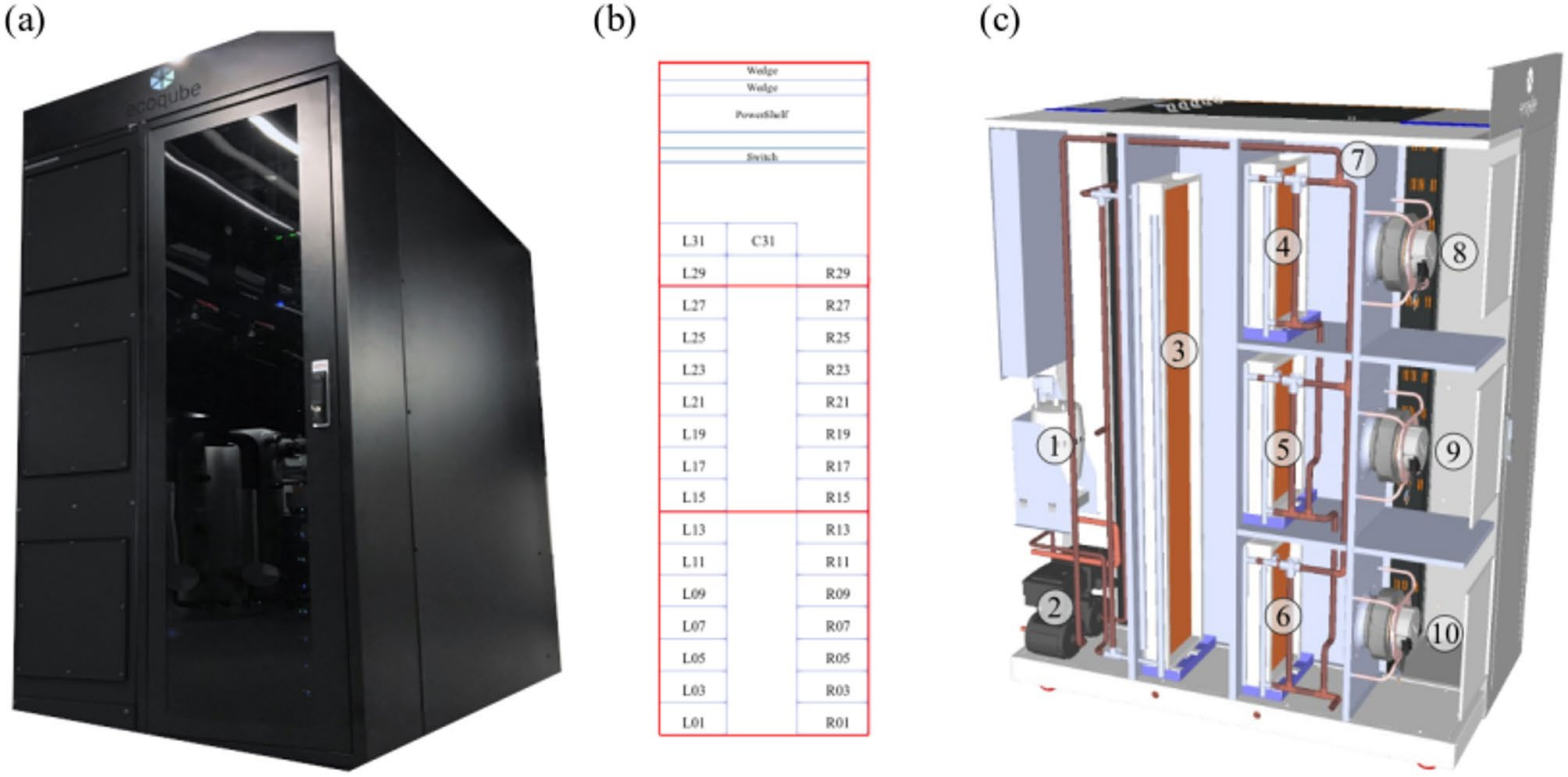
(**a**) Snapshot of the edge data center (**b**) rack layout and (**c**) three-dimensional view of the cooling system.

The NEST (Next Evolution in Sustainable Building Technologies) demonstrator, a modular research and innovation building operated by Empa in Switzerland, hosts a pilot data center to simulate IT scenarios in European Union-funded projects ECO-Qube^40^ and HEATWISE^41^. It is hosted in a mixed-use vertical energy district that can be configured flexibly to emulate different use cases in terms of cooling technologies and waste heat utilization. The NEST hosts an open hardware, single rack, air-cooled micro data center which runs commercial IT loads of up to 12 kW and stores data. The micro data center shown in Fig. 1(a) is integrated into the thermal and electricity grids of the NEST building. NEST’s active thermal grid, which incorporates a variety of thermal grids interconnected by thermal storage, heat exchangers and heat pumps, provides a platform to explore waste heat capture under diverse IT loads. As shown in Fig. 1(b), the rack cabinet contains 32 identical Leopard V3.1 models of Open Compute Project (OCP) servers, wedge units, and power shelves. The servers are numbered sequentially from bottom to top and labeled as left and right regions on the figure.

As shown in Fig. 1(c), the hot air exhausted from the IT equipment is cooled by the cooling coils integrated into the medium temperature grid (MTG) and low temperature grid (LTG) to regulate various flow regimes within the building. The heat is transferred from the hot air to the water through the coils and the cooled air is directed by the fans to the inlets of the servers. The LTG comprises of three water coils, which can be controlled by three-way valves mounted at the inlet of each coil to distribute water supplied by the pump. The hot water from the LTG is directed to the cooler of the building as a cooling load, while the water heated by the MTG is directed to the building as waste heat recovered from the data center. Components of the cooling system shown in Fig. 1(c) are also listed in Table 1. The cooling system illustrated in Fig. 1(c) was designed and manufactured to address the specific needs of the corresponding projects^40,41^.

**Table 1:** Components of the cooling unit.

Component	Description
1	Humidifier
2	Low and medium temperature grid pumps
3	MTG coils
4,5,6	LTG coils
7	Valve
8,9,10	Fans

The IT and cooling data within the data center are managed through an integration of several data monitoring platforms such as Prometheus^42^, Grafana^43^ and Modbus^44^. Prometheus is utilized to monitor and store Intelligent Platform Management Interface (IPMI) data from the IT equipment. Parameters of the cooling system such as valve opening ratio (VOR) and fan speed (FS), are adjusted via Modbus to control water flow rate through the coils and regulate fan speeds. Finally, Grafana^43^ is employed to visualize the sensor data collected from the cooling unit.

### Experimental characterization of the cooling system

Experimental studies were conducted at the pilot data center by adjusting the VOR and FS of the cooling system using the Modbus protocol to model flow and thermal characteristics of the cooling system in the multi-region numerical model.

### Modeling flow rate of water

**Fig. 2:**
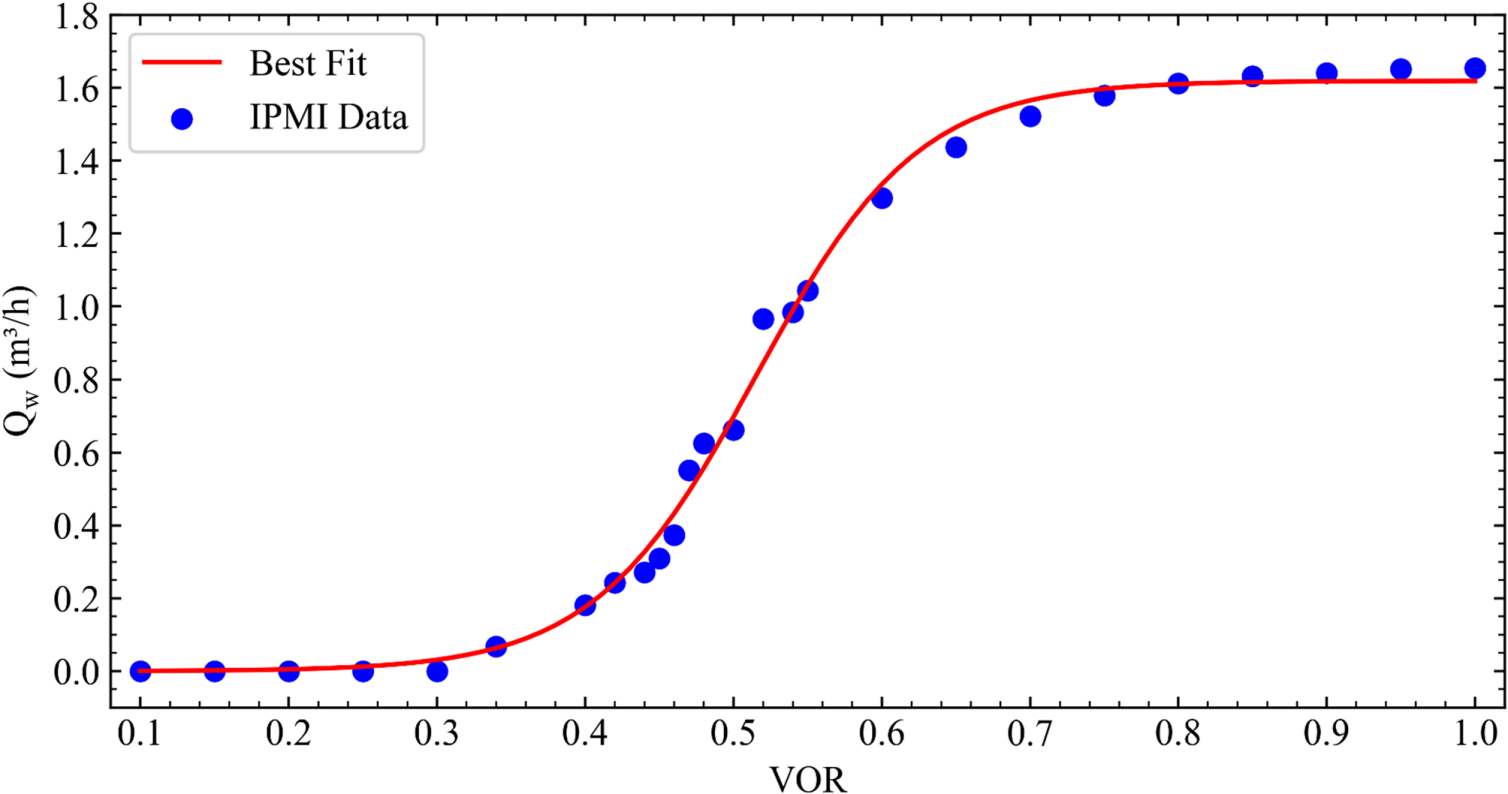
Best fit to the measured water flow rates for various VORs.

Flow rate of water ($\:{Q}_{w}$) passing through the coils is controlled by the VOR parameter using the Modbus protocol. Experimental tests were conducted for various VOR values with the FS set to 5% to observe variations of the flow rate as a function of VOR itself. Data were recorded during 15 min for each VOR and time-averaged values of the recorded flow rates are plotted with respect to the VOR in Fig. 2. The flow rate of water through the coils remained nearly uninfluenced by the valve opening at both low and high settings. Based on the recorded data, the variation in the flow rate of water with VOR is described by the following sigmoid function:

1 $$\:{Q}_{w}=\frac{a}{1+b{e}^{-c\left(VOR-d\right)}}$$

where $\:{Q}_{w}$ is the flow rate of water, empirical coefficients are determined as *a* = 1.61934, *b* = 0.370234, *c* = 18.2585 and *d* = 0.569614 with $\:{R}^{2}$=0.995. The VOR can be determined from the following equation explicitly to adjust the $\:{Q}_{w}$ during experimental tests:

2 $$\:VOR=\frac{-ln\left(\frac{1.61934-{Q}_{w}}{0.370234{e}^{10.4177}}\right)}{18.2585}$$

### Modeling flow rate of air

The flow rate of air supplied by the fans of the cooling system is typically measured using flow meters located at specific positions of DCs^45,46^. However, uncertainties arising from the combined effects of measurement location, the number of flow meters and alteration of air flow caused by the presence of the measurement devices in such a small medium may reduce reliability of the measured data. In this study, instead of using flow meters, the airflow rate is estimated from the following energy balance equation for the coil:

3 $$\:\rho_{a}C{p}_{a}{Q}_{a}$$

**Fig. 3:**
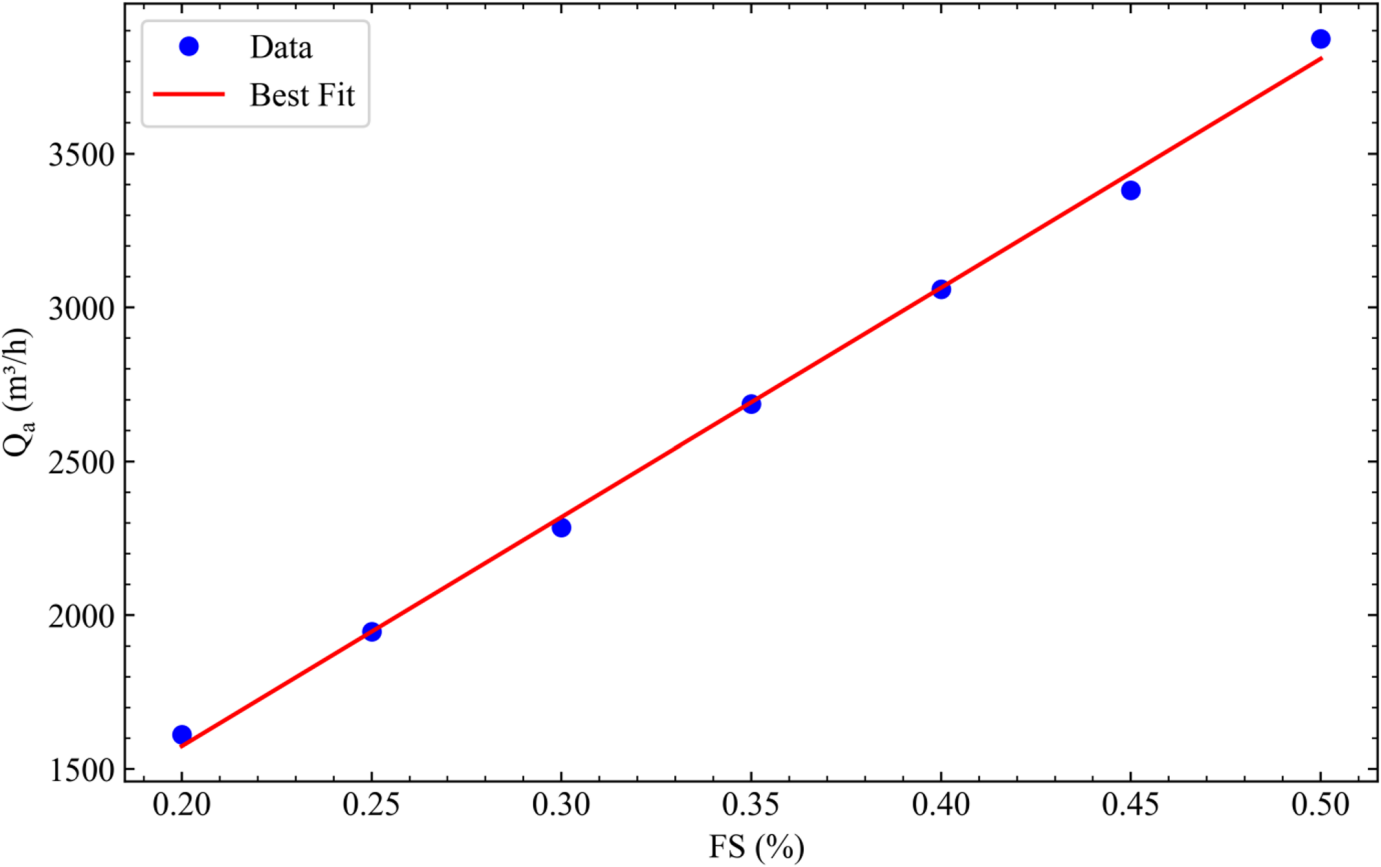
Variation of flow rate of air with FS.

Where $\:{T}_{a,\in\:}$ and $\:{T}_{a,out}$ are air temperatures at the inlet and outlet of the coil, respectively, $\:{T}_{w,\in\:}$ and $\:{T}_{w,out}$ are water temperatures at the inlet and outlet of the coil, respectively. Among these parameters, the $\:{T}_{a,\in\:}$, $\:{T}_{a,out}$, $\:{Q}_{w}$, $\:{T}_{w,\in\:}$ and $\:{T}_{w,out}$ can be monitored via Grafana. Experimental studies were conducted for various FS and VOR values to estimate air flow rate considering pressure losses by internal components shown Fig. 1c and to see the possible effect of the VOR on the results. As can be seen in the time-averaged values in Table 2, effect of the VOR is trivial on the results. Therefore, average values given in the last column were plotted in Fig. 3 and used in the data analysis. The linear relation between flow rate and FS can be correlated with the following empirical equation with $\:{R}^{2}$=0.997:

4 $$\:{Q}_{a}=7448.857xIFS+84.508$$

Where $$\:{Q}_{a}$$ in m^3^/h.

**Table 2:** Measured airflow rates for various FS and VOR.

Fan Speed (V)	Q_a_ (m^3^/h)				
	VOR = 50%	VOR = 60%	VOR = 70%	VOR = 80%	Average
20	1656.35	1631.00	1611.08	1546.22	1611.16
25	1921.41	1923.79	1895.86	2044.43	1946.37
30	2323.48	3211.41	2225.76	2280.46	2510.28
35	2671.99	2767.18	2678.88	2624.81	2685.72
40	3180.07	2952.13	2954.41	3148.49	3058.78
45	3487.07	3412.28	3294.93	3328.89	3380.79
50	4067.03	3835.92	3824.37	3765.46	3873.20

### Modeling heat transfer through cooling coils

Effectiveness is crucial for the estimation of the heat transfer through the cooling coils based on the flow rates of air and water. Effectiveness of the heat exchanger can be described by the following equation:

5 $$\:\epsilon\:=\frac{{P}_{Coil}}{{P}_{max}}$$

Where $\:{P}_{Coil}$ is the power of the coil that can be monitored via the Grafana and $\:{P}_{max}$ is the maximum amount of energy that can be removed from air using the following equation:

6 $$\:{P}_{max}={C}_{min}$$

**Fig. 4:**
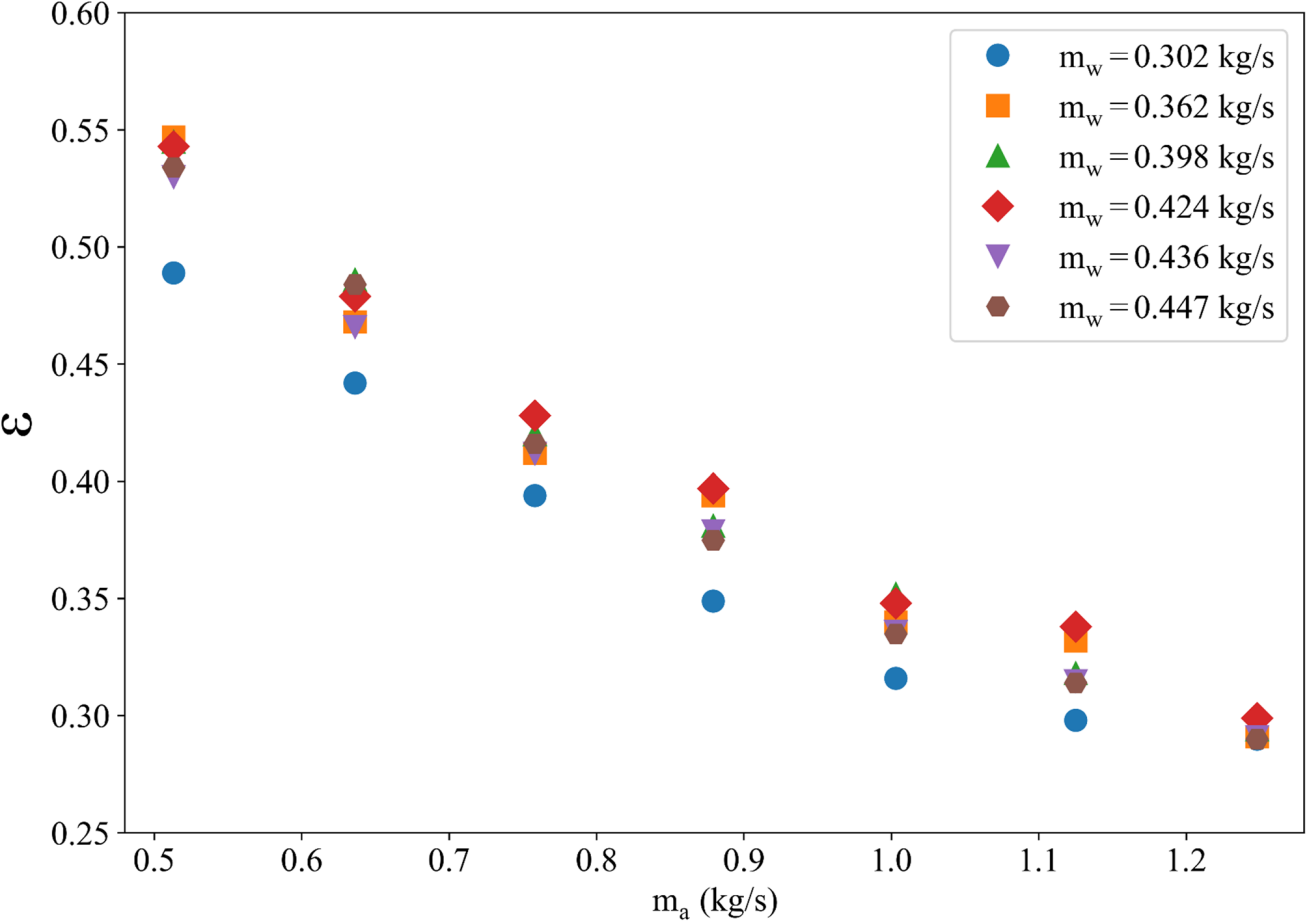
Effectiveness curves for the cooling coil.

Where $\:{C}_{min}=min\left({C}_{a},{C}_{w}\right)$, $\:{C}_{a}={\rho}_{a}.C{p}_{a}.{Q}_{a}$ and $\:{C}_{w}={\rho}_{w}.C{p}_{w}.{Q}_{w}$. Subscripts *a* and *w* in the equations represent air and water, respectively, $\:C$ is the specific heat capacity and $\rho$ is the fluid density. Experimental studies were conducted systematically using 49 conditions as combinations of various VOR and FS values to obtain effectiveness table for the present cooling coil. The VOR values changed between 50% and 80% and the FS values changed between 20% and 50% during experimental studies. Figure 4 shows variation of the effectiveness with air and water mass fluxes. The generated effectiveness table can be used in the numerical model for the realistic simulation of the heat transfer through the coils.

The present CHT model employs the effectiveness-NTU method for modeling heat transfer through the cooling coils^47^:

If $$\:{C}_{a}<{C}_{w}$$:

7 $$\:NTU=-\left(\frac{1}{{C}_{r}}\right)ln\left[{C}_{r}ln\left(1-\epsilon\:\right)+1\right]$$

if $$\:{C}_{a}\ge\:{C}_{w}$$:

8 $$\:NTU=-ln\left[1+\left(\frac{1}{{C}_{r}}\right)ln\left(1-\epsilon\:{C}_{r}\right)\right]$$

$$\:W\text{h}\text{e}\text{r}\text{e},\:{C}_{r}={C}_{max}/{C}_{min}\:and\:{C}_{max}=max\left({C}_{a},{C}_{w}\right).$$.

### Fast evaluation of thermal field

Numerical models provide detailed and accurate analyses of airflow and temperature distributions, enabling precise simulation of various cooling strategies and their effects on data center performance^48–50^. However, CFD simulations are computationally intensive and time-consuming, making them less practical during optimization studies. To address this challenge, the FTE approach described in^37^ is used in the present study for the fast prediction of inlet and outlet temperatures of servers under a given IT load distribution. In this model, the relationship between inlet and outlet temperatures of a server $\:i$ is characterized by the following energy equation:

9 $$\:{P}_{i}={{\rho}}_{a}{f}_{i}{C}_{p,a}\left({T}_{i}^{out}-{T}_{i}^{}\right)$$

**Fig. 5:**
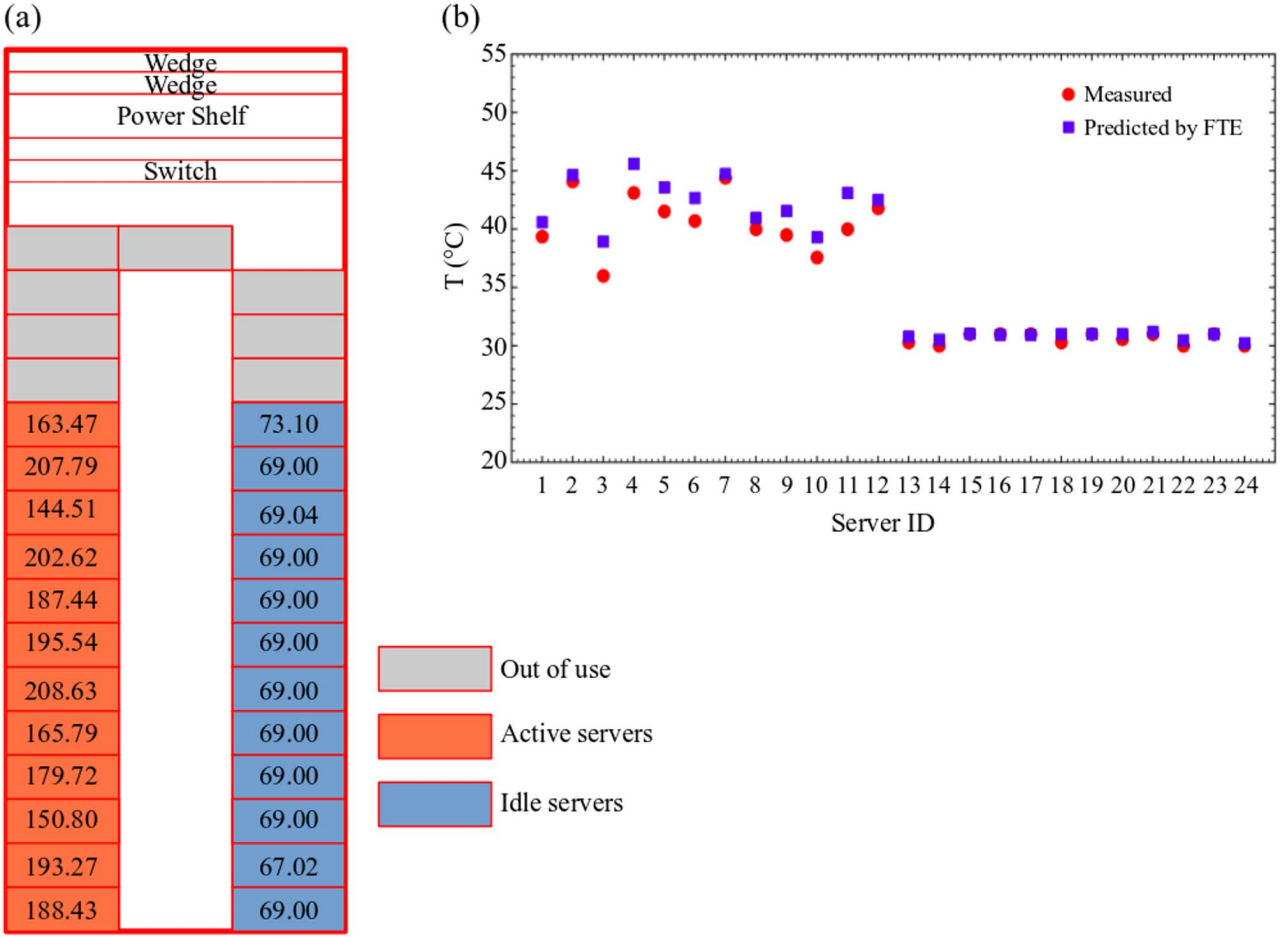
(**a**) Distribution of server power consumptions over the rack layout and (**b**) comparison of measured and predicted outlet temperatures.

Where $\:{f}_{i}$ is the flow rate of air passing through the server, $\:{\rho}_{a}$ is the air density and $\:{C}_{p,a}$ is the specific heat of air. In this study, 24 simulations were performed for profiling, and one simulation was performed for the referencing stage to model the $\:{P}_{i}$ matrix in Eq. (9), which represents interaction between servers considering recirculation effects. The working scenario shown in Fig. 5(a) was designed for the validation of the FTE approach in the present data center. Workloads were generated by running CFD jobs using parallel computing at different utilization rates, resulting in power consumption levels shown in Fig. 5(a), and allocated to the data center using the SLURM workload manager. Workloads were not submitted to the servers represented by grey since these servers were allocated for data storage and management. Servers on the right side remained in an idle condition. Outlet temperatures of servers were monitored and recorded via Prometheus during the experimental study. Figure 5(b) compares the measured and predicted outlet temperatures. Mean and maximum prediction errors were determined to be 1.23% and 5.76%, respectively. A statistical significance analysis was conducted to assess performance of the prediction model. The present FTE model achieved an RMSE of 1.44 °C and an mean absolute error of 1.09 °C, with a coefficient of determination of R²=0.93, indicating a strong correlation with the experimental data. A paired t-test indicated a statistically significant difference between predicted and measured values (t = 5.25, *p* = 2.5 × 10^−5^< 0.001). The mean bias was calculated as 1.06 °C with a 95% confidence interval of [0.64, 1.48] °C. The Bland–Altman analysis further showed that the maximum difference lays within the limits of agreement (− 0.84–2.97 °C). These results indicate that the FTE approach used in the present study can accurately predict outlet temperatures of servers for a given workload distribution.

### Numerical modeling of data center

#### Governing equations

Accurate modeling of heat transfer between air and water can be achieved through a multi-region CHT model. Continuity, momentum and energy equations are solved sequentially in air and water regions considering turbulence and buoyancy effects. Compressible and turbulent flow can be represented by the following continuity and momentum Eq^51^.:

10 $$\:\frac{\partial\:{\rho}}{\partial\:t}+\frac{\partial\:{\rho}{u}_{j}}{\partial\:{x}_{j}}=0$$

11 $$\:\frac{\partial\:}{\partial\:t}\left({\rho}{u}_{i}\right)+\frac{\partial\:}{\partial\:{x}_{j}}\left({\rho}{u}_{i}{u}_{j}\right)=\frac{-\partial\:p{\prime\:}}{\partial\:{x}_{i}}+\frac{\partial\:}{\partial\:{x}_{j}}\left(\mu\:\frac{\partial\:{u}_{i}}{\partial\:{x}_{j}}\right)+\frac{\partial\:}{\partial\:{x}_{j}}\left(-{\rho}{u}_{i}{\prime\:}{u}_{j}{\prime\:}\right)$$

Where $\rho$ is the density, $\:t$ is the time, $\:{u}_{i}$ is the velocity component in the $\:{x}_{i}$ direction, $\:p{\prime\:}$ is the modified pressure and $\:\mu\:$ is the molecular viscosity. The turbulent stress in Eq. (11) can be calculated from the Boussinesq approximation:

12 $$\:-{\rho}{u}_{i}{\prime\:}{u}_{j}{\prime\:}={\mu\:}_{t}\left(\frac{\partial\:{u}_{i}}{\partial\:{x}_{j}}+\frac{\partial\:{u}_{i}}{\partial\:{x}_{i}}\right)-\frac{2}{3}k{\delta\:}_{ij}$$

Where $\:k$ is the turbulent kinetic energy and $\:{\delta\:}_{ij}$ is the Knocker delta. In this study, the $\:k-\omega\:$ SST turbulence closure model is employed to simulate turbulence effects in the air region. Flow is laminar and incompressible in water region due to the low Reynolds number flow through the coils. Two transport equations are solved for the kinetic energy $\:k$ and specific dissipation rate $\:\omega\:$^52^. Then, turbulent viscosity can be calculated from the following equation in the air region:

13 $$\:{\mu\:}_{t}=\frac{{\rho}{a}_{1}k}{max\left({a}_{1}\omega\:,SF2\right)}$$

Where $\:S$ is the strain rate invariant and $\:{a}_{1}$ is the model constant^51^. The energy equation is solved for the flow^52^:

14 $$\:\frac{\partial\:}{\partial\:t}\left({\rho}E\right)+\frac{\partial\:}{\partial\:{x}_{i}}\left[{u}_{i}\left({\rho}E+p\right)\right]=\frac{\partial\:}{\partial\:{x}_{j}}\left[{\lambda\:}_{eff}\frac{\partial\:{T}_{f}}{\partial\:{x}_{j}}+{u}_{i}\frac{\partial\:}{\partial\:{u}_{j}}\left(\mu\:\frac{\partial\:{u}_{i}}{\partial\:{x}_{i}}-{\rho}{u}_{i}{\prime\:}{u}_{j}{\prime\:}\right)\right]+{S}_{E}$$

Where $\:E$ is the total energy and $\:{S}_{E}$ is the volumetric heat source to represent power generating components on the servers in the numerical model. Thermo-physical properties such as density and specific heat capacity are calculated dynamically according to the temperature field in the numerical model^53,54^.

Energy equations are formulated and solved for air and water separately to model heat transfer between two distinct fluid regions. At the interface, coupled boundary conditions are imposed to ensure continuity between heat flux and temperature. This approach accurately captures the interaction between two fluids, maintaining consistency with the principles of energy conservation^46,47,55^. Temperature fields in air and water regions are calculated using the following equation:

15 $$\:\rho\:{C}_{p}\left(\frac{\partial\:T}{\partial\:t}+{u}_{i}\frac{\partial\:T}{\partial\:{x}_{i}}\right)=\frac{\partial\:}{\partial\:{x}_{j}}\left(k\frac{\partial\:T}{\partial\:{x}_{j}}\right)+{S}_{i}$$

Where $\:\rho\:$ denotes the fluid density, $\:{C}_{p}$ is the specific heat of the fluid, $\:u$ is the flow velocity, $\:T$ is the temperature, $\:k$ is the thermal conductivity and $\:{S}_{i}$ represents the volumetric heat source. Radiation effects are not considered in the present CHT model. In addition, cooling coils are modeled as porous regions and heat transfer within these regions is simulated using the effectiveness-NTU method.

Continuity of temperature ($\:{T}_{a}={T}_{w}$) and heat flux ($\:-{k}_{a}\frac{\partial\:{T}_{a}}{\partial\:n}=-{k}_{w}\frac{\partial\:{T}_{w}}{\partial\:n}$) must be maintained on the boundary between two fluid regions to ensure energy conservation and accurate representation of heat transfer process.

#### Mesh and boundary conditions

Numerical simulations were performed using the open-source CFD software OpenFoam-v2312, which employs a finite volume approach to discretize governing equations over a control volume using PIMPLE. This algorithm combines PISO (Pressure Implicit with Splitting of Operator) and SIMPLE (Semi-Implicit Method for Pressure-Linked Equations) algorithms for pressure-velocity coupling. Convective and gradient terms in the air region were discretized using bounded Gauss upwind and Gauss linear schemes, respectively, while Laplacian terms were discretized using a limited corrected Gauss linear method with a limiting coefficient of 0.33. In the water region, convective terms were discretized using the bounded Gauss upwind method and Laplacian terms were discretized using a Gauss linear uncorrected scheme. Discretized equations are stored in upper, lower and diagonal matrices in the tridiagonal data storage framework of OpenFoam. The Geometric-Algebraic MultiGrid (GAMG) and Preconditioned Bi-Conjugate Gradient Stabilized (PBiCGStab) linear solvers were used for solving pressure and momentum equations, respectively, to optimize performance in parallel computing.

**Fig. 6:**
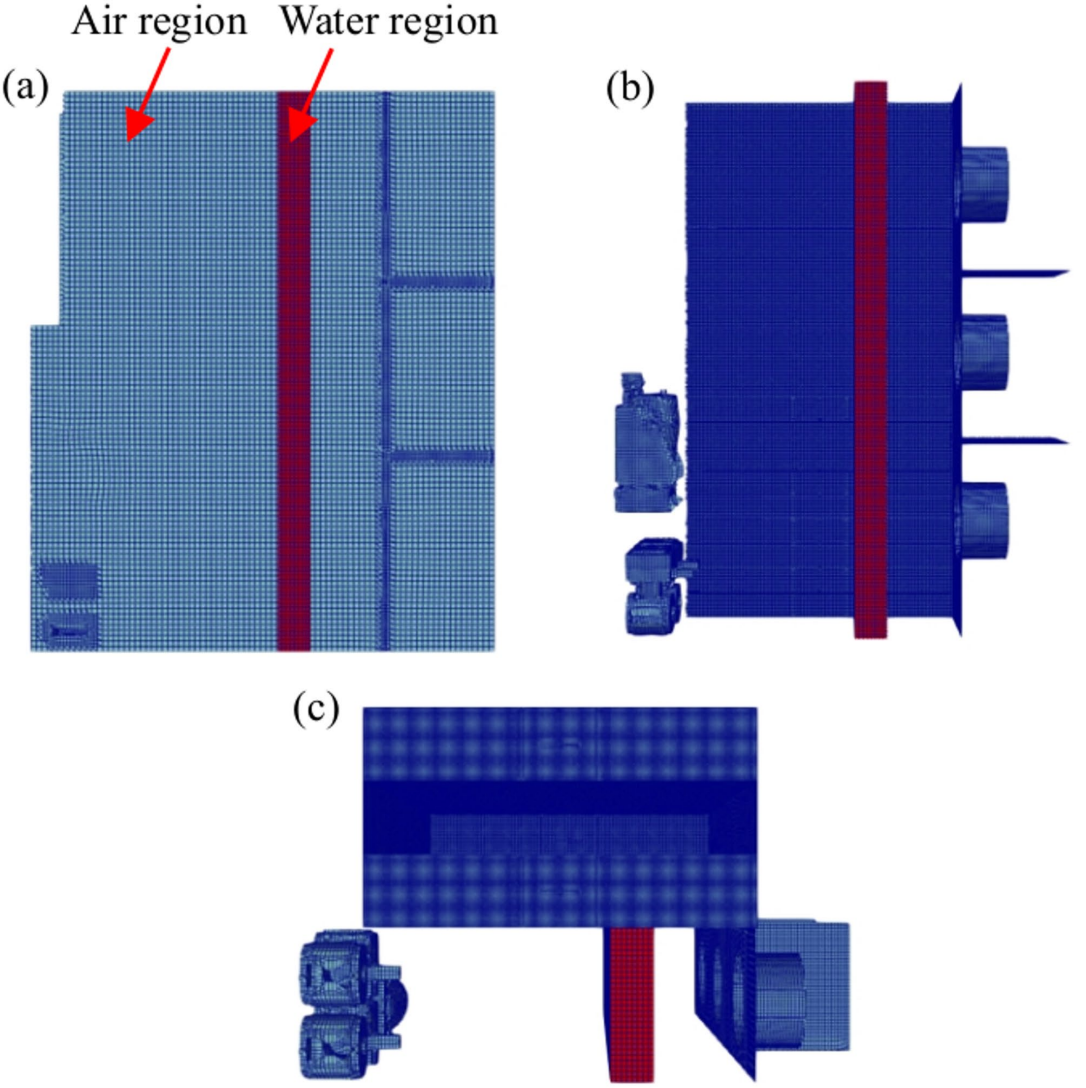
Right views of the (**a**) internal mesh and (**b**) internal components (**c**) top view of the internal components.

In the present CHT model, cooling coils are modeled as water region and the remaining part of the data center is modeled as air region. A block-structured mesh was generated in the water region using the blockMesh utility. In the air region, the mesh was generated using the snappyHexMesh utility with both hexahedral and split-hexahedral meshing techniques in parallel computing. An advanced hex-dominant meshing algorithm was applied to snap internal solid objects (Fig. 1c) and to ensure a high-quality mesh with a minimal skewness and non-orthogonality. The multi-region mesh generated for the present micro data center is shown in Fig. 6. The mesh statistics listed in Table 3 indicate that the present mesh is suitable to accurately approximate convective and diffusive fluxes on cell faces.

**Table 3:** Mesh statistics.

Region	Number of Cells	Maximum Skewness	Maximum Non-orthogonality	Minimum Volume	Maximum Volume
Air	3,150,557	8.42	66.15	2.09e-08	1.35e-05
Water	31,136	1.20e-13	0	3.27e-06	3.28e-06

## Results and discussion

In this section, numerical model is validated with the experimental measurements. Then, the MOTA is introduced and demonstrated using the validated numerical model in terms of cooling load on the cooling system of the building and waste heat recovery from the micro data center.

### Validation of the CHT model

**Fig. 7:**
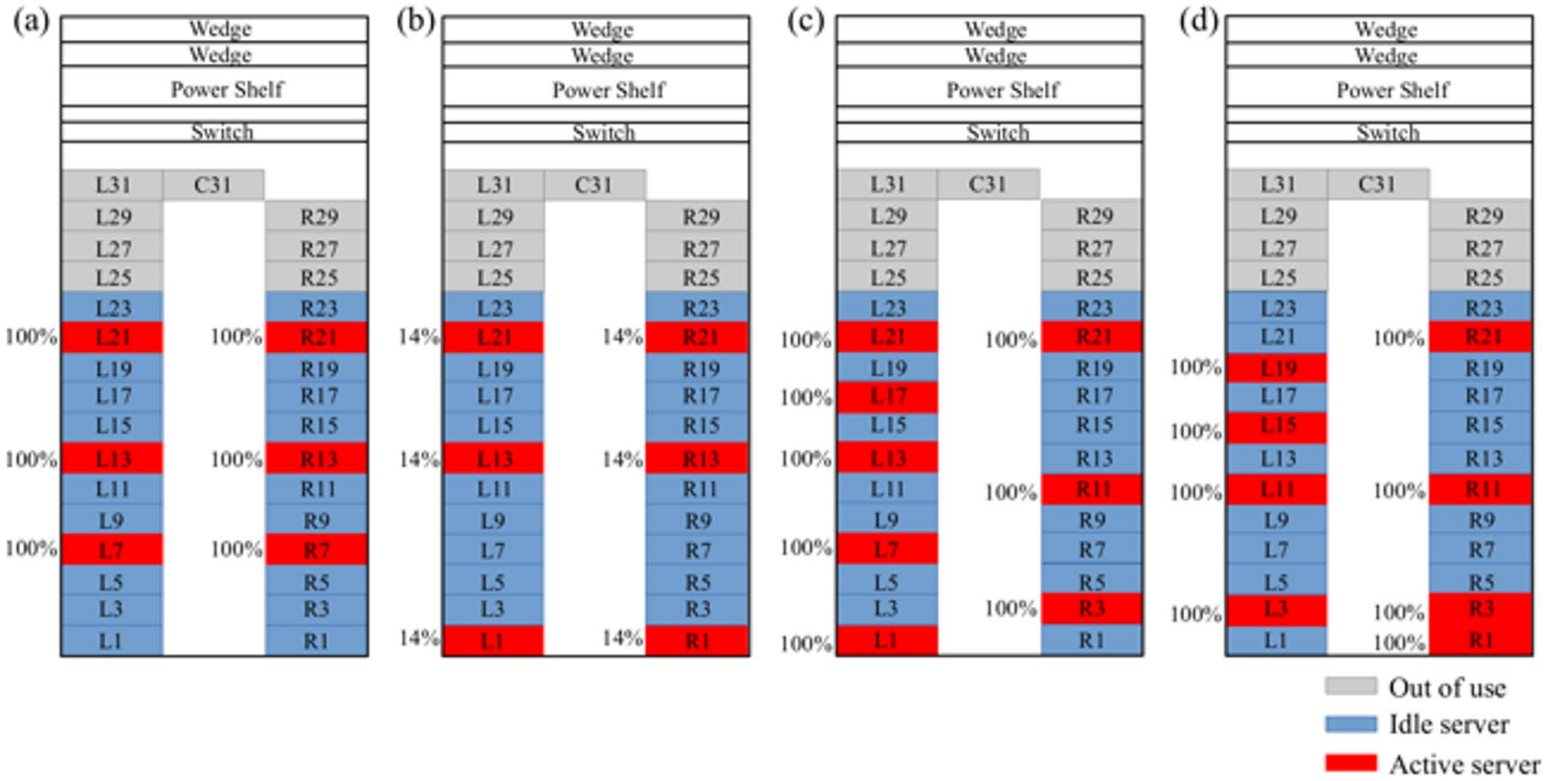
Workload distributions and utilization rates: (**a**) Test 1 (FS = 30% and VOR = 55%), (**b**) Test 2 (FS = 30% and VOR = 55%), (**c**) Test 3 (FS = 30% and VOR = 50%) and (**d**) Test 4 (FS = 30% and VOR = 55%).

Four experimental tests were conducted using various utilization rates and workload distributions shown in Fig. 7 to demonstrate accuracy and reliability of the present numerical model under diverse conditions in terms of workload distribution, total IT load and cooling settings. The FS and VOR parameters of the cooling system were adjusted to ensure reliable cooling of the environment under a given working scenario. The IPMI data were recorded during 15 min at each test and time-averaged values of the recorded data were used for the validation of the numerical model. The active servers, indicated in red in Fig. 7, were utilized 14% and 100% during experimental tests.

The FS and VOR parameters of the cooling system, provided in the caption of Fig. 7, were used in the numerical simulations based on the empirical Eqs. (2) and (4), respectively. Water temperatures measured at the inlet of the cooling coil were applied as boundary conditions in the numerical model. Measured and simulated air temperatures at the inlet and outlet of the coil, as well as the water temperature at the outlet of the coil, are compared in Table 4. The consistency observed between measured and simulated air temperatures demonstrates that the present thermal model predicts temperature distribution at the outlet of the servers accurately. The maximum error of 10.37% between measured and simulated water temperatures at the outlet of the coil indicates that the present heat transfer model, as implemented in the cooling system, can be used reliably. The present CHT model successfully predicts the reduction in air temperature at the coil due to the heat exchange between air and water.

**Table 4:** Comparison of numerical and experimental results.

Test	Temperature	Experimental (°C)	Numerical (°C)	Error (%)
Test 1 (3 kW)	$\:{T}_{w,out}$	15.16	14.28	5.80
	$\:{T}_{a,\in\:}$	27.64	27.13	1.85
	$\:{T}_{a,out}$	20.86	19.76	5.26
Test 2 (2.2 kW)	$\:{T}_{w,out}$	15.06	13.95	7.36
	$\:{T}_{a,\in\:}$	26.59	24.92	6.30
	$\:{T}_{a,out}$	20.62	18.48	10.37
Test 3 (3.5 kW)	$\:{T}_{w,out}$	16.14	15.59	3.42
	$\:{T}_{a,\in\:}$	28.47	29.31	2.96
	$\:{T}_{a,out}$	22.06	20.49	7.10
Test 4 (3.5 kW)	$\:{T}_{w,out}$	15.29	14.38	5.98
	$\:{T}_{a,\in\:}$	27.60	27.52	0.27
	$\:{T}_{a,out}$	21.02	19.90	5.31

**Fig. 8:**
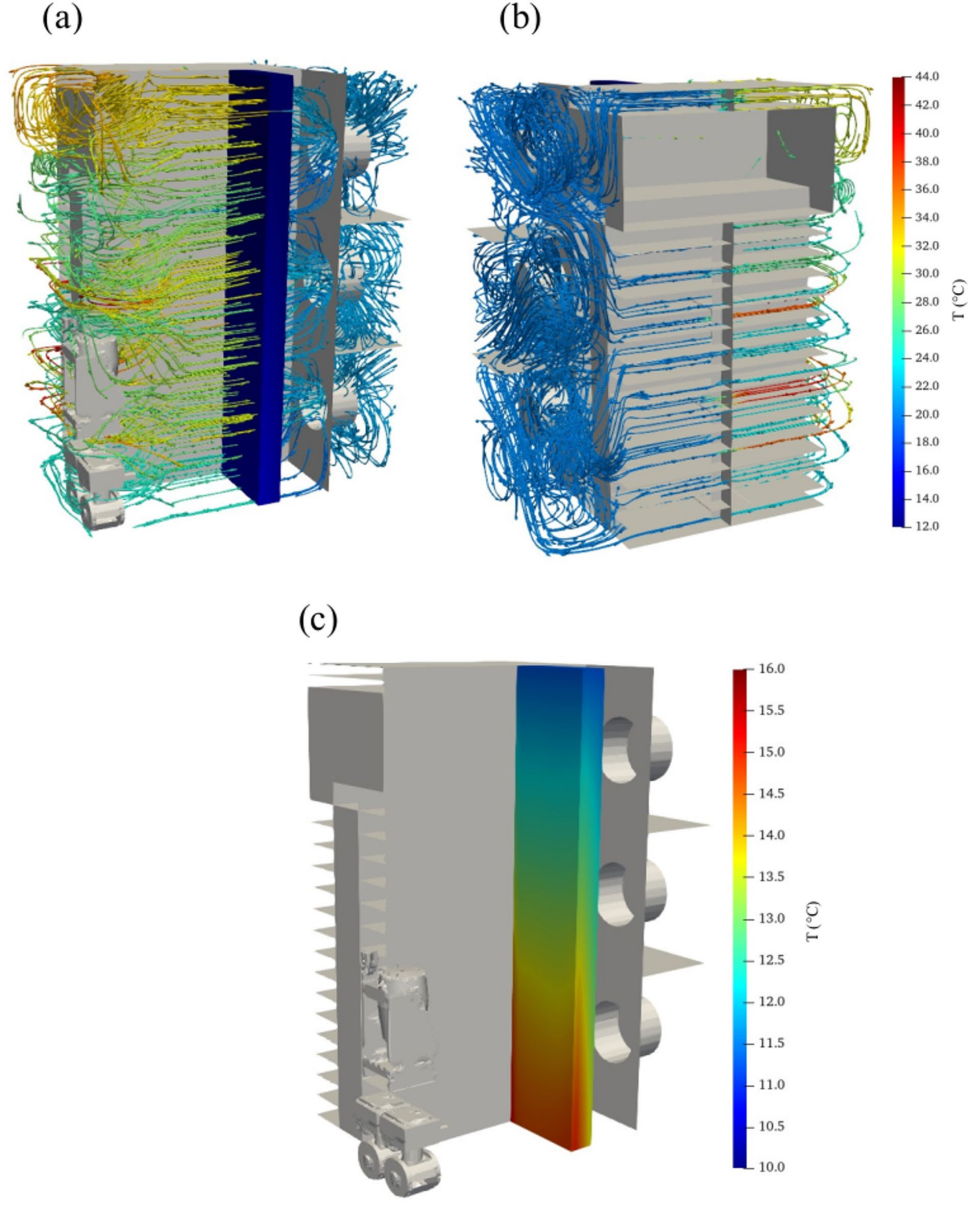
(**a**) Right view and (**b**) left view of the flow structures and (**c**) temperature distribution over the LTG under the working scenario of Test 1 (FS = 30% and VOR = 55%).

As visualized in Fig. 8(a), the air heated by the servers cools down as it flows through the LTG due to the thermal energy transfer. The cooled air is then directed to the server inlets by fans of the cooling unit to recirculate airflow inside the airtight micro data center. In Fig. 8(b), it is evident that the cold air directed to the server inlets absorbs heat from the heat sources defined in active servers. Subsequently, the heated air travels through the server outlets and leaves the rack cabinet. The highest temperatures within the data center are observed near the servers since heat sources are defined in the servers to represent CPUs as heat sources. Figure 8(c) provides a detailed view of a variable temperature distribution over the LTG. Within this grid, a significant heat exchange occurs at the interface where air meets water. As the water flows toward the coil outlet, a noticeable increase in its temperature is observed, indicating the effectiveness of heat transfer in this region.

The present CHT model was validated against four experimental cases from the testbed, which were selected to represent a diverse range of IT loads and cooling boundary conditions. The good agreement observed between simulation results and measurements across these cases supports the reliability of the model under operational conditions of the pilot data center.

### Development of the MOTA

Numerical simulations are performed using various VOR values of LTG and MTG under the working scenario shown in Fig. 7(a) to see effect of the water flow rate on the cooling load and waste heat recovery performance. The simulated *P*_*Coil*_ and *P*_*MTG*_ values listed in Table 5 show that the cooling load can be minimized and waste recovery can be maximized when the *VOR*_*LTG*_ and *VOR*_*MTG*_ are selected as 50% and 65%, respectively. Therefore, this cooling configuration is preferred during the optimization of workloads.

**Table 5:** Variations of cooling load and recovered heat with VOR values.

Case	VOR_LTG_ (%)	VOR_MTG_ (%)	*P*_Coil_ (kW)	*P*_MTG_ (kW)
Case-1	50	50	1.30	2.36
Case-2	50	65	1.13	2.47
Case-3	50	80	1.11	2.45
Case-4	65	50	1.36	2.29
Case-5	65	65	1.21	2.42
Case-6	65	80	1.19	2.41
Case-7	80	50	1.34	2.31
Case-8	80	65	1.20	2.41
Case-9	80	80	1.18	2.39

**Fig. 9:**
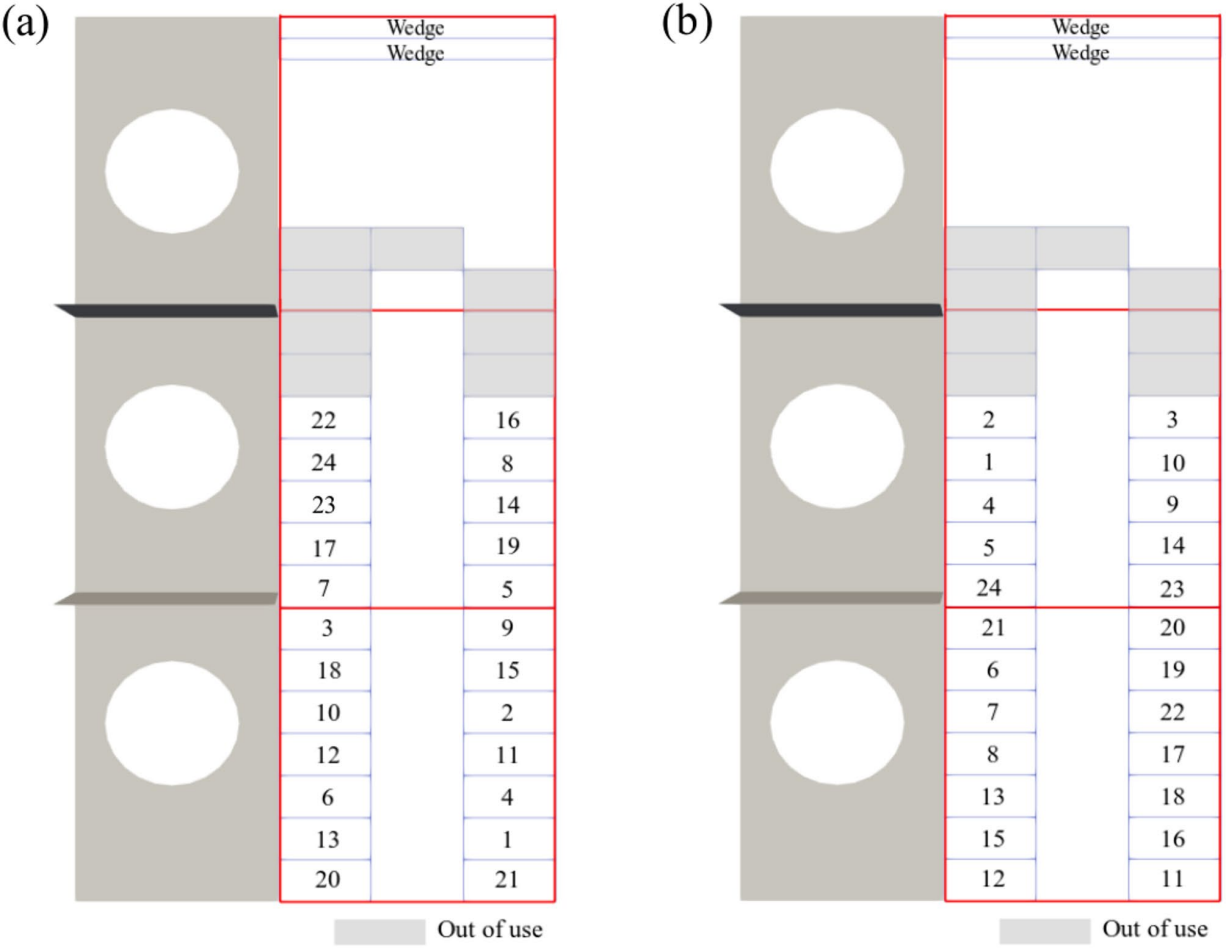
Allocation of workloads sequentially using (**a**) Random and (**b**) MOTA approaches.

Although servers in the present data center are identical, their exposure to varying degrees of cold or hot air varies based on their locations, resulting in distinct heating and cooling dynamics. Locations of IT equipment are closely related to the airflow patterns, server fan types, and the cooling infrastructure. Consequently, even when a fixed workload is assigned to each server, CPU temperatures fluctuate according to the aforementioned thermal factors. The objective of workload distribution optimization is to utilize spatial characteristics of IT equipment to maximize waste heat recovery. To this end, the FTE Model^37,38^ is used to calculate outlet temperatures of servers under an assigned IT load distribution. These outlet temperatures are then directly fed into the MTG within the cooling unit. The MOTA employs the FTE method to optimize workload distributions and maximize server outlet temperatures under the constraints outlined by ASHRAE^12^. This algorithm strategically allocates server workloads to maximize average rack outlet temperature, thereby improving waste heat recovery. Figure 9 illustrates working principles of two approaches, where a power consumption of 300 W is sequentially assigned to an available server using Random and MOTA workload assignment algorithms. The numbers on the servers represent sequence of the assigned work. A total IT load of 7.2 kW is assigned to the data center at the end of the assignment process.

**Fig. 10:**
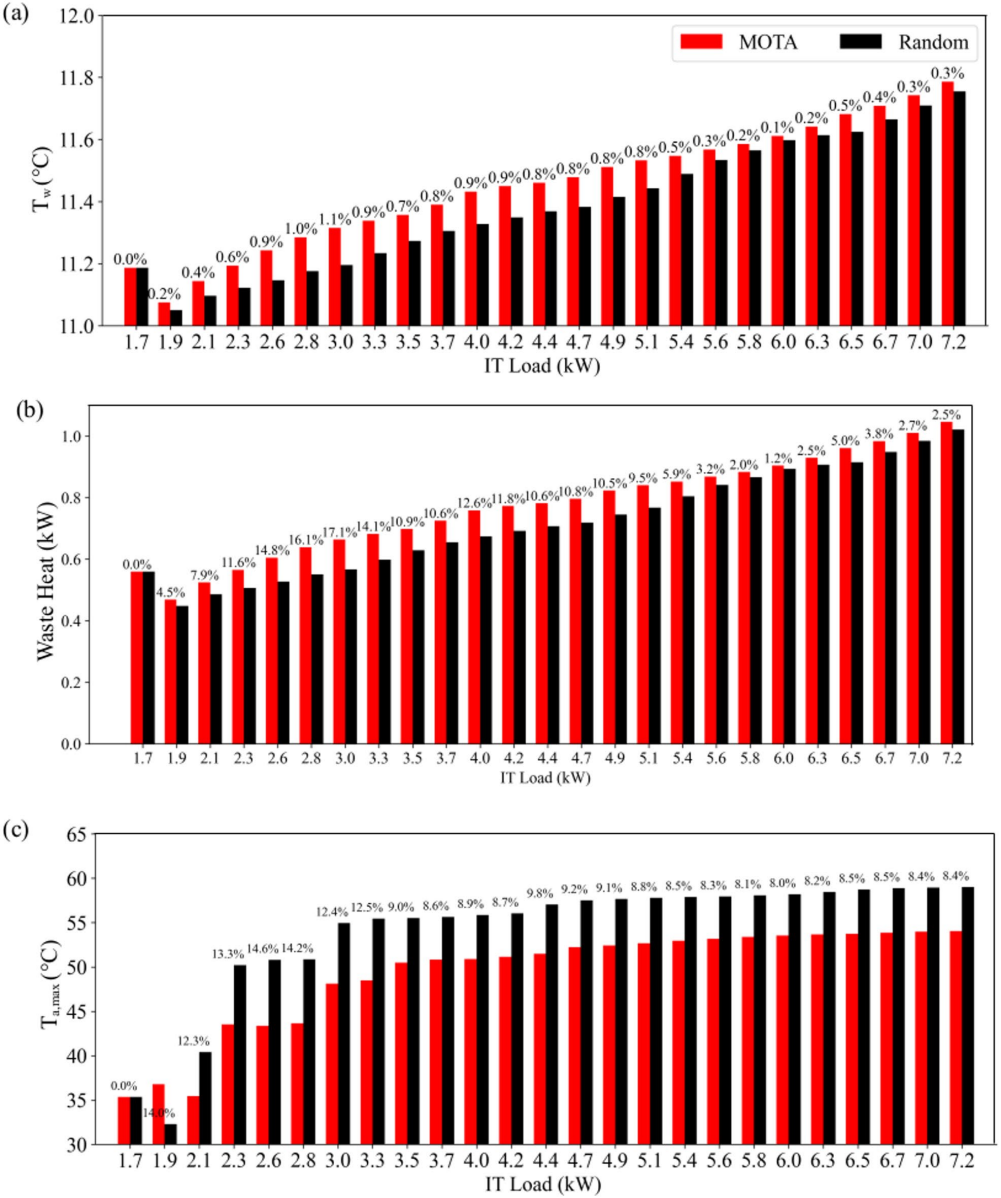
Comparison of the (**a**) volume average water temperature in the MTG, (**b**) waste heat recovery from the MTG and (**c**) maximum air temperature with difference percentage between MOTA and random IT distributions.

Figure 10(a) compares volumetric average water temperatures in the MTG when workloads are assigned randomly and when using the MOTA. The water temperature rises continuously as new workloads are assigned to the data center. However, the temperature difference between two approaches tends to decrease with increasing workload and converges to an identical value when the rack is fully utilized. Figure 10(b) compares waste heat recovery from the data center when the workloads are assigned according to the Random and MOTA approaches. The MOTA increases waste heat recovery by 17.1% compared to the Random approach when the IT load is 3 kW. As shown in Fig. 10(c), the load transferred to the cooling system of the building was reduced by 14.6% when the workloads were assigned using the MOTA. The proposed approach also maintains its superiority over the randomly distributed workload assignment algorithm achieving at least an 8% improvement even when the data center capacity exceeds 50%.

**Fig. 11:**
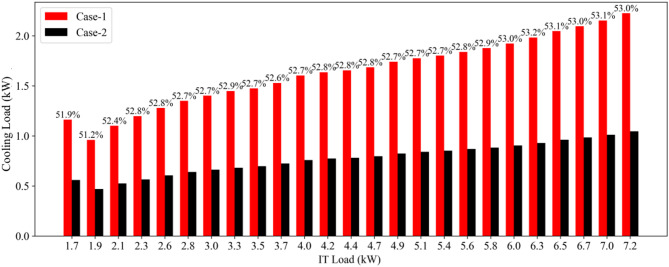
Variations of cooling load with the IT load for various flow rates.

To demonstrate the impact of water flow rate on the cooling load as a function of the IT load, the numerical simulations were repeated using the cooling configuration of Case 1 in Table 5 and results are compared in Fig. 11. The significant difference observed between two cooling settings tends to increase with the IT load, highlighting the importance of optimizing cooling parameters in a data center based on workloads.

**Fig. 12:**
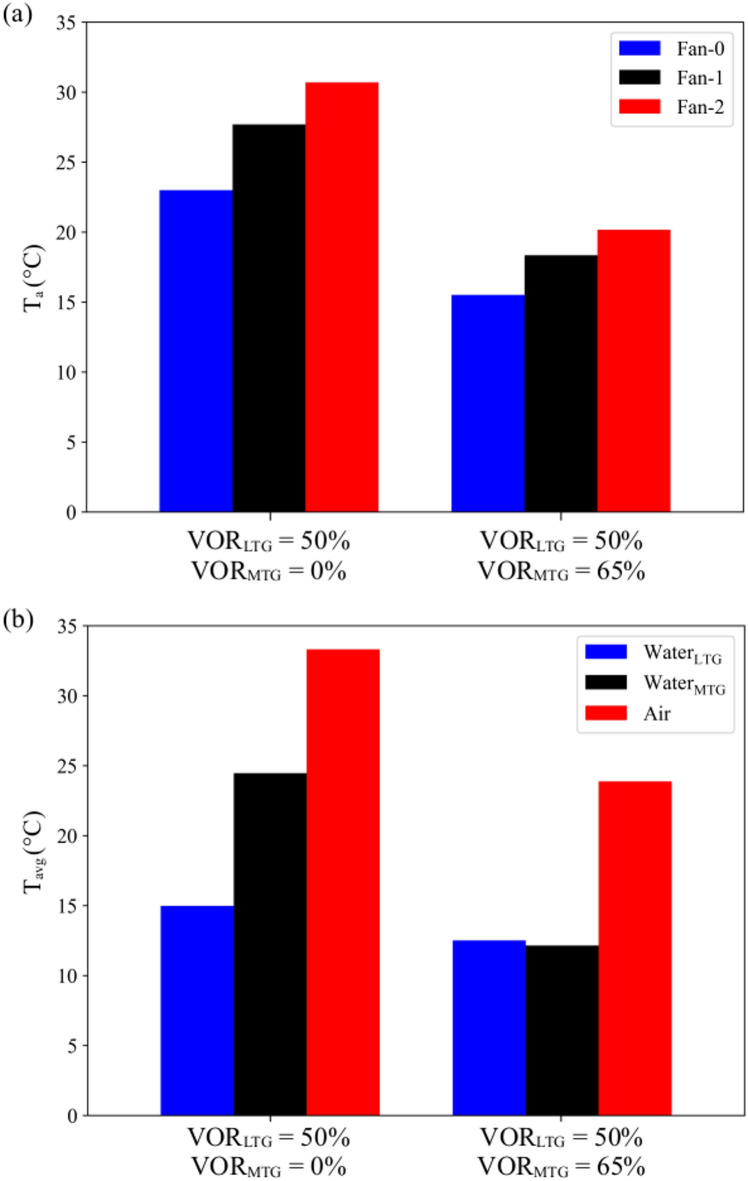
(**a**) Air temperatures at the outlet of the cooling fans and (**b**) volumetric average temperatures at air and water regions for P_IT_=7.2 kW.

Numerical simulations were conducted with the valve of the MTG closed (VOR_MTG_=0). The exhaust heat from the servers was captured by the LTG and transferred to the cooling system of the building. The VOR values of the LTG and MTG were selected as the optimal values determined from the numerical experiments. Maximum air temperatures at the outlet of the fans are compared in Fig. 12(a). When the MTG is activated, the maximum air temperature is reduced by 34.3% because a portion of the heat is removed from the air by the MTG. As shown in Fig. 12(b), the volumetric average of water temperature LTG is reduced by 16.4% when the MTG is activated, which highlights a significant reduction in the cooling load of the building. Activation of the MTG also reduces the average air temperature within the data center. The improvement achieved by MOTA tends to decrease as the total IT load increases due to fact that servers operate close to their maximum utilization and the flexibility to strategically reallocate or redistribute workloads reduces as thermal and performance characteristics across servers become uniformly constrained On the other hand, under low IT loads, the MOTA can allocate workloads to servers thermally advantageous positions, thereby achieving more pronounced gains in outlet temperature management and waste-heat recovery compared to traditional workload algorithms.

A variety of thermal-aware and energy-efficient workload allocation strategies have been proposed in the literature, including sensor-based fast thermal evaluation^56–58^, thermal-aware scheduling^59–61^ and chip-temperature-based allocation^39^. These methods aim to reduce local hotspots and minimize IT energy consumption. However, their optimization objectives and application layers differ fundamentally from the present approach, where the MOTA targets maximization of server outlet temperatures to maximize waste heat recovery efficiency at the building integration level. Furthermore, implementing chip- or inlet-level balancing algorithms was not feasible within the constraints of the pilot testbed, which operated on uniform OCP servers integrated into a building thermal grid. Therefore, we used random allocation as the baseline case to demonstrate impact of the proposed approach in terms of waste heat recovery. The MOTA integrates a validated multi-region CHT model with an FTE framework to maximize waste heat recovery at the building integration level. Unlike conventional thermal-aware scheduling methods that focus on balancing chips or inlet temperatures, the MOTA directly optimizes server outlet temperatures, which are the critical drivers for improving heat exchanger effectiveness and increasing return water temperature. This design enables not only more accurate prediction of thermal interactions within the rack but also tangible gains in waste heat utilization for secondary energy use in buildings.

### Real-world applicability, computational cost and limitations

The MOTA improves waste-heat recovery and reduces cooling load, but its deployment in real-world systems may involve trade-offs due to facts that some jobs could experience slight slowdowns, hardware may be exposed to greater thermal cycling, and network traffic can increase if workloads are not co-located. These risks can be mitigated by limiting workload migrations, favoring rack-local placement, and operating MOTA as an advisory layer to existing schedulers, thereby preserving thermal gains while protecting performance, service-level agreements, and equipment reliability.

The present study was conducted on a single-rack micro data center with homogeneous air-cooled servers under fixed experimental conditions. While this configuration provided a controlled environment to demonstrate feasibility, the MOTA is also applicable to larger and more diverse facilities, including those with heterogeneous server clusters and latency-sensitive workloads. In such environments, workload allocation must jointly consider thermal efficiency, performance heterogeneity, and service level agreement compliance. Extending MOTA to these scenarios may require multi-objective optimization frameworks, integration of predictive models of heterogeneous server behavior, and coupling with cooling models that account for liquid-based technologies. Future work will focus on adapting and validating the algorithm under such broader configurations to ensure scalability and practical applicability across different data center architectures.

All numerical simulations in this study were performed using the open-source CFD software OpenFOAM. The multi-region CHT model developed for air–water heat transfer was executed with parallel computing on servers located at the Empa Data Center. These servers are equipped with dual-socket Intel^®^ Xeon^®^ E5-2680 v4 processors (2.40 GHz), providing 56 logical CPUs (2 × 28 cores with hyper-threading enabled) and 70 MB shared L3 cache, supported by 256 GB DDR4 RAM. Approximately 4 GB of memory was utilized during CHT runs. The runtime overhead of the MOTA workload allocation algorithm was negligible compared to the computational demand of the CFD simulations, with allocation decisions completed within a few seconds on this hardware.

## Conclusion

In this study, a novel approach was proposed for the maximization of waste heat recovery from a building integrated edge data center. For a given workload, the proposed MOTA approach can optimize workload distribution using the FTE method to ensure that the outlet temperature of each server is maximized within the temperature limits outlined by the standards. To demonstrate performance of the proposed approach compared to the conventional workload assignment strategy, a CHT model was developed in which flow and thermal characteristics of air and water regions are simulated separately, and the heat exchange between air and water is calculated based on the energy balance without using empirical heat transfer coefficients. Cooling coils were modeled as porous regions in the multi-region numerical model. Parameters of the cooling system were determined from the experimental studies and implemented into the numerical model to enable realistic simulations under various cooling settings in the present framework. A comparison of simulation results with the experimental measurements under distinct IT and cooling scenarios confirms that the CHT model can reliably calculate air temperatures within the data center and water temperatures at the coils. Following main conclusions are drawn from this study:

1. Compared with the randomly distributed workloads, the MOTA can improve waste heat recovery by up to 17.1% and reduce the cooling load transferred to the cooling system by up to 53.2%.
2. The MOTA effectively increases return temperatures and reduces the energy required for additional temperature boosts via heat pumps for domestic use. A higher return air temperature expands the temperature differential between supply and return air. This broader temperature difference improves cooling unit efficiency to remove more heat per unit of airflow.
3. The cooling system presented in this study offers a potential pathway for waste heat recovery from air-cooled servers in edge data centers integrated with buildings.

Simulations performed using a validated numerical model on a pilot data center demonstrate the potential of the proposed tool for energy-efficient management of data centers within building energy systems and reducing environmental footprint of data centers. This study is developed and demonstrated at a specific micro data center configuration with predefined cooling system parameters and workload distributions. Future studies should therefore focus on extending the applicability of the proposed methodology to larger-scale and heterogeneous data center environments.

The validation was performed on a single-rack, homogeneous, air-cooled micro data center under fixed operating conditions. Broader applicability to large-scale and heterogeneous server clusters, as well as to alternative cooling technologies such as direct-to-chip liquid cooling, require further investigation. Additionally, the runtime and computational overhead of the MOTA should be evaluated in more detail to confirm its suitability for latency-sensitive edge computing environments. Future work will therefore focus on extending the methodology to diverse hardware configurations and cooling technologies, integrating multi-objective optimization to jointly consider thermal, energy and performance metrics.

## Data Availability

All data generated or analysed during this study are included in this published article or available from the corresponding author on reasonable request.

## List of symbols

A': Cross-interference matrix
C_p_: Specific heat capacity (J/kgK)
C_pa_: Specific heat capacity of air (J/kgK)
C_pw_: Specific heat capacity of water (J/kgK)
$$\delta_{ij}$$: Knocker delta
E: Total energy (J)
ε: Effectiveness of the heat exchanger
f_i_: Air flow rate in the i-th server (CFM)
g_i_: Gravitational acceleration in the i-direction (m/s^2^)
h: Favre-averaged enthalpy (J)
tttk: Turbulent Kinetic Energy (m^2^/s^2^)
m_a_: Mass flux of the air (kg/s)
m_w_: Mass flux of the water (kg/s)
${\mu},\mu_t$: Molecular and turbulent viscosities (m^2^/s)
ω: Specific dissipation rate (s^−1^)
Q: Flow rate (m^3^/s)
Q_a_: Flow rate of air (m^3^/s)
Q_w_: Flow rate of water (m^3^/s)
Q^i^_in_: Inlet flow rate of server (m^3^/s)
Q^i^_out_: Outlet flow rate of server (m^3^/s)
P_MTG_: Waste heat (W)
P_max_: Maximum amount of energy (W)
P_new_: New power consumption distribution matrix
P_ref_: Reference power consumption distribution matrix
$\rho_a$: Density of air (kg/m^3^)
$\rho_w$: Density of water (kg/m^3^)
S_i_: Momentum source
S_E_: Source term
T: Temperature (°C)
T_a,in_: Air temperature at the inlet of the low temperature grid (°C)
T_a,out_: Air temperature at the outlet of the low temperature grid (°C)
T_w,in_: Water temperature at the inlet of the low temperature grid (°C)
T_w,out_: Water temperature at the outlet of the low temperature grid (°C)
$T^{new}_{out}$: New outlet temperature distribution matrix
$T^{ref}_{out}$: Reference outlet temperature distribution matrix
T^i^_in_: Inlet temperature of the server (°C)
T^i^_out_: Outlet temperature of the server (°C)
T_sup_: Supply temperature (°C)
U: Velocity (m/s)

## Abbreviations

CHT: Conjugate heat transfer
DC: Data center
FTE: Fast thermal evaluation
GAMG: Generalized Geometric-Algebraic MultiGrid
HPC: High Performance Computing
IT: Information technology
IBM: International Business Machines
ICT: Information and Communication Technologies
FS: Fan speed
IPMI: Intelligent Platform Management Interface
kW: Kilowatt
LTG: Low temperature grid
MTG: Medium temperature grid
MOTA: Maximization of outlet temperatures algorithm
NTU: Number of Transfer Units
OCP: Open Compute Project
PBiCGStab: Stabilised preconditioned bi-conjugate gradient
PIMPLE: Combination of PISO and SIMPLE
PISO: Pressure Implicit with Splitting of Operator
SIMPLE: Semi-Implicit Method for Pressure-Linked Equations
VOR: Valve opening ratio
WS: Working scenario
